# Efficacy of an Ayurvedic Management Protocol on the Rate of Recurrence, Healing and Safety in Parikarthika (Fissure in Ano): Protocol for a Randomized Controlled Trial

**DOI:** 10.2196/88548

**Published:** 2026-07-08

**Authors:** Indu P P, Kalpana Kachare, Pratap Shankar K M, Binitha P, Lisha S, Bhagwan Sahai Sharma, Thugutla Maheswar, Arunabh Tripathi, Narayanan Srikanth, Rabinarayanan Acharya

**Affiliations:** 1 National Ayurveda Research Institute for Panchakarma, Central Council for Research in Ayurvedic Science, Cheruthuruthy, Thrissur, Kerala, India; 2 Central Council for Research in Ayurvedic Science, 61-65 opp. D' Block, D Block, Janakpuri Institutional Area, Janakpuri, New Delhi, Delhi, 110058, India, 91 9834410028; 3 Regional Ayurveda Research Institute, Central Council for Research in Ayurvedic Science, Poojappura, Thiruvananthapuram, Kerala, India

**Keywords:** parikarthika, fissure in ano, anal infiltration, jatyadi taila, recurrence, safety, healing.

## Abstract

**Background:**

*Parikarthika* refers to the condition in which the individual experiences a sensation of pain as if the *Guda* is being cut around with scissors. This condition can be correlated to the fissure in ano. It has been described as an acute superficial break in the continuity of the anoderm, usually in the posterior midline of the anal canal. The incidence of anal fissures is around 1 in 350 adults. Management can be divided into conservative, operative, and adjuvant therapies. The main challenge in the management of fissure in ano is the increased rate of recurrence. Here comes the importance of an alternative therapy which can reduce the recurrence. The recurrence rate of fissure in ano by nonsurgical procedures is 60%, and with the *Ayurveda* protocol, the expected recurrence rate is 30%.

**Objective:**

This study aims to evaluate the efficacy of an *Ayurvedic* management protocol on the rate of recurrence, healing, and safety in *Parikarthika* (fissure in ano).

**Methods:**

A prospective, randomized controlled interventional open-labeled study has been designed to enroll a total of 90 participants from the OPD (Outpatient Department) of National Ayurveda Research Institute for Panchakarma (NARIP), Cheruthuruthy, Kerala. They will be screened for fissure in ano based on physical examination. Participants satisfying the inclusion and exclusion criteria will be randomly allocated to either the trial group (n=45) or the control group (n=45). In the trial group, participants will be managed with *Avipattikar churna* and *Triphala guggulu* internally and *Jatyadi taila* anal infiltration, as well as *Lepana* along with sitz bath in *Triphala kashaya* externally. Diltiazem gel will be given for external application in the control group along with *Triphala kashaya* sitz bath and *Triphala churna* internally. The intervention is given for a period of 28 days, and participants are evaluated on 14th, 28th, 56th, 84th, and 112^th^day. The assessment parameters include recurrence rate, treatment efficacy, safety, comparative tolerability, and change in health-related quality of life.

**Results:**

This project was funded in June 2024, and the study duration is 36 months. The first participant was enrolled in April 2025; 9 participants were enrolled as of May 2025. The data analysis will be completed by October 2027 and the results are anticipated to be published by December 2027.

**Conclusions:**

The study is expected to demonstrate the efficacy of an *Ayurvedic* management protocol on the rate of recurrence, healing, and safety in *Parikarthika* (fissure in ano).

## Introduction

### Background

Fissure in ano, commonly referred to as *Parikarthika* in *Ayurveda,* is a longitudinal split in the anoderm of the distal anal canal, which extends from the anal verge proximally towards but not beyond the dentate line [1]. The incidence of anal fissures is around 1 in 350 adults [2]. They are equally common in men and women, and most often occur in adults aged 15 to 40. The lifetime incidence of anal fissures is estimated to be around 11%, with males and females equally at risk [3]. In a recent study from India, the prevalence of anal fissures among adult patients with anorectal problems was reported to be 17.8%, indicating that every sixth patient with any anorectal problem may have anal fissures as the underlying pathology [4]. Overstretching of the epithelial lining of the anal canal due to hard feces, ischemia, sphincter hypertonia, direct injury caused by surgical procedures like haemorrhoidectomy, repeated childbirth, underlying pathologies like Crohn’s disease, ulcerative colitis, tuberculosis or sexually transmitted diseases, and laxative abuse were considered as general etiological factors [5]. In women, the occurrence of fissure in ano is commonly associated with menstruation, pregnancy, and childbirth trauma. Pain during and after defecation, bleeding as streaks along with the stools, changed bowel habits, and burning pain in the anal region are the most common presentations in fissure in ano. A fissure wound typically heals on its own within approximately six weeks and is classified as acute. However, if the fissure persists beyond six weeks, it is termed a chronic fissure in ano [6]. In chronic cases, pain tends to be less severe but more persistent, and sentinel pile development may occur at the anal verge. It may require more extensive treatment due to its resistance to healing with conservative methods.

Generally, the management of fissure in ano can be divided into conservative, operative, and adjuvant therapy. Despite advances in diagnostic and therapeutic approaches, recurrence of fissures in ano remains a significant concern of today. The recurrence rate of anal fissure can be quite high, with studies reporting a recurrence rate of up to 40% of patients, meaning a significant chance of the fissure coming back even after initial healing; this is particularly true for chronic anal fissures where the recurrence rate can be even higher, sometimes reaching 80% or more [7]. Recurrence rate of anal fissure is influenced by several factors like choice of treatment, patient compliance, underlying comorbidities, and lifestyle modifications. Nonoperative management generally possesses a recurrence rate of 18.6% and a healing rate of 50%. According to studies, topical nitrates exhibit a 1% rate of late recurrence and a 68% healing rate by about 8 weeks, whereas topical Diltiazem application shows a 65% recurrence rate with fewer side effects. Botulinum toxin injection is one of the widely used nonoperative therapies for fissure in ano, which carries a recurrence rate of 42% and 60%‐80% healing rate. Patients with fissures that are chronic and not manageable with the above-said methods were advised to undergo operative procedures. Among the operative techniques, lateral sphincterotomy is considered the gold standard; it has a 90%‐97% healing rate with a 2%‐5% risk of fecal incontinence. The recurrence rate after lateral sphincterotomy varies between 1.6% and 6 %. Inadequate sphincterotomy, chronic morphological changes of the internal anal sphincter, poor bowel habits, or other medical conditions such as ulcerative colitis and Crohn disease can increase the recurrence of fissure in ano. It is clear that even after surgery, anal fissures can recur, and complications can arise [8]. Furthermore, treating recurrent fissures postsurgically presents challenges and a less favorable prognosis. This necessitates further research into alternative approaches that offer improved efficacy, reduced recurrence, and enhanced safety.

In accordance with the Ayurvedic classic literature, fissure in ano can be compared to *Parikarthika* based on its clinical presentation. *Parikarthika* is characterized by *Karthanavath* or *Chedanavath Soola* (sharp shooting pain) in *Guda pradesa. Kshatapayu* and *Kshata guda* are the synonyms of this condition [9]. *Ayurveda* does not consider it as an independent disease but as a complication of *Virechana* and *Vasthi karm*a. As per *Acharya Charaka* and *Susruta* in the context of *Virechana vyapath,* it is mentioned that if a person with *Mridu koshta* and *Alpabala* ingests *Thikshna*, *Ushna* and *Ruksha* drugs for *Virechana* will cause *Parikarthika*. Susruta acharya considers it *vatapaithik*a in nature and characterized by burning pain over *Guda, Nabhi, Medra,* and *Vasthisira* associated with arrest of flatulence, abdominal distention, and tastelessness of food [10]. As per *Acharya Charaka,* excruciating pain, mild bleeding, and discharge are the symptoms of *Parikarthika* [11]. While detailing *Vasthivyapath, Acharya Charaka* said that administration of *Ruksha, Thikshna Vasthi* in excess, to a person with *Mridukoshta* and *Alpa dosha* vitiation will result in *Parikarthika*. Pain over *Trika, Vamkshana, and Adhonabhi* are its peculiar features as per *Acharya Charaka* [11]. Inappropriate insertion and defect in *Vastinetra* itself may cause this disease. Apart from these, *Charaka* and *Vagbhata Acharyas* have mentioned *Parikarthika* as a symptom of *Vatajaatisara,* attributing its occurrence to trauma caused by stools [10]. As per *Acharya Charaka* in *Sidhisthana, Parikarthika* is described as a complication of the excessive use of *Yapanavasti* [11]. *Kasyapa* considers *Parikarthika* as a disease of gravid women, which is characterized by cutting and tearing pain in *Guda pradesa* with the involvement of the three *doshas* [12].

In *Ayurveda, Parikarthika Chikithsa* solemnly depends on the cause. Generally, the treatment procedures can be summarized as local measures and gastroenterological systemic correction as per classical textbooks. In addition to conservative management, parasurgical measures such as *Ksharasuthra* and *Agnikarma* can be adopted for chronic conditions [13,14]. The local measures comprise *Guda abhyanga, Guda parisheka, Picha vasthi,* and *Anuvasana vasthi*, whereas systemic correction can be achieved by following concepts such as *Langhana, Langhana Pachana,* and *Brimhaneeya Vidhi* in accordance with the stage of disease and *Bala* of the patient. *Vata hara Ahara*, milk diet is specifically mentioned in this context. Numerous scientific studies have been carried out across various academic and research institutions, including CCRAS (Central Council for Research in Ayurvedic Sciences), to evaluate and substantiate the efficacy of Ayurvedic approaches in the management of *Parikarthika,* viz. local application of *Jatyadighrita, Jatyaditaila, Anu taila, Prabhakara ghrita, Kasisadi Taila, Nimbaditaila, Vranaropanataila, Doorvadighrita, Vedanantaka Malahara,* and internal administration of *Abhyarishta, Kutjarista, TriphalaChurna*c have demonstrated superior efficacy compared to conventional therapies in managing the signs and symptoms [15].

Despite advances in diagnostic and therapeutic approaches, the recurrence in fissure in ano remains a significant concern today. The recurrence rate of anal fissure is influenced by several factors like choice of treatment, patient compliance, underlying comorbidities, and lifestyle modifications. Metabolic correction and dietetic regulations are unique approaches in *Ayurveda*. This underscores the significance of alternative therapy in reducing the recurrence rate. This study highlights the efficacy of an *Ayurvedic* management protocolfor fissures in ano, focusing on its impact on recurrence rates, safety, and healing. Understanding their efficacy and applicability can pave the way for more comprehensive and sustainable management options for this challenging condition.

### Study Objective

The primary objective of the study is to evaluate the recurrence rate of *Parikarthika* after the management with ayurvedic management protocol in comparison with Diltiazem topical application. The secondary objective is to study the efficacy of the ayurvedic management protocol for healing *Parikarthika* (fissure in ano), in comparison with Diltiazem topical application and to study the safety of the Ayurvedic management protocol for *Parikarthika* (fissure in ano), in comparison with Diltiazem topical application. Assessment of comparative tolerability and change in health-related quality of life will also be served as the secondary objective of the trial.

## Methods

### Design

This study is a single-center, prospective, interventional, open-labeled randomized controlled trial (RCT), evaluating the efficacy of an *Ayurvedic* management protocol on rate of recurrence, safety, and healing in *Parikarthika* (fissure in ano).

### Study Setting

Eligible participants would be identified at the Outpatient and Inpatient departments of the National Ayurveda Research Institute for Panchakarma (NARIP),

### Study Population

Individuals with chronic anal fissure will be randomly selected from the OPD of NARIP regardless of their sex, socio-economic status, and other parameters, but fully satisfying the inclusion criteria.

### Diagnostic Criteria

Clinical diagnosis of chronic anal fissure (ie, fissures persisting for longer than 6 wk) of primary or typical occurring in the posterior or anterior midline, characterized by an indurated margin and a base consisting of either scar tissue or sphincter muscle fibers, with or without sentinel tag, presenting with clinical features like pain on defecation, bright red bleeding, or constipation. The inclusion and exclusion criteria are shown in (Textbox 1).

Textbox 1. **Inclusion and exclusion criteria.**

**Inclusion criteria**:

- Individuals of either sex with age between 18 and 60 years
- Individuals diagnosed of parikarthika with pain, bleeding per rectum and constipation
- Individuals, willing to give written informed consent to participate in the study

**Exclusion criteria:**

- Individuals who are also suffering from, hemorrhoids, fistula in ano, peri anal abscess, fecal incontinence, and anal stenosis
- Acute fissure in ano, secondary fissures, fissures located at lateral location and multiple fissures
- Individuals with comorbidity such as diabetes mellitus, uncontrolled Hypertension, inflammatory bowel disease, sexually transmitted diseases, Hepatitis B and C, tuberculous ulcer, clinically significant renal disease, hepatic disease, cardiovascular disease and psychological diseases
- Individuals on active drug abuse, alcoholics and on medication hampering wound healing
- Known sensitivity to any of the trial drugs or their ingredients
- Individuals who are already underwent operative treatment and under non operative treatments like botulinum injections or calcium channel blockers or topical nitrates
- Individuals who are currently undergoing either of the trial intervention within 30 days of randomization of the study
- Pregnant/lactating women
- Any other condition which the Investigator thinks may jeopardize the study

### Withdrawal Criteria

It must be emphasized that participation is voluntary and participants have the right to opt out of the trial at any time without any prejudice. Withdrawal from the study may also occur due to any major ailment necessitating the new modalities of treatment or noncompliance with treatment. If the relationship of a suspected ADR (adverse drug reaction),/AE (adverse event),/SAE (severe adverse event) with the trial intervention is confirmed by causality assessment, the individual will be withdrawn from the clinical trial. In addition, if individuals meet any exclusion criteria (whether newly developed or previously unrecognized) or if they choose to withdraw from the study, they will also be considered withdrawn.

### Participant Identification

The individual suffering from chronic anal fissure will be randomly selected from the OPD of NARIP Cheruthuruthy regardless of their sex or socioeconomic status, but fully satisfying the inclusion, exclusion, and diagnostic criteria. Patients will be personally informed about the research project and will receive written information. Written informed consent will be obtained from the participant. Only participants who have given informed consent will be enrolled in this study. The participants will be randomly allocated to either the trial group (n=45) or the control group (n=45).

### Randomization, Blinding, and Allocation Concealment

Block randomization with unequal block sizes will assign participants to either group 1 or group 2 in a 1:1 ratio. “Sealed Envelope” software is used to generate the random number sequences. An independent statistician not involved in the participant’s enrollment and assessment will generate the randomization sequence. Sequentially numbered opaque sealed envelopes (SNOSE) will be used to ensure allocation concealment. The participants’ enrolment number will be printed at the top of the envelope, and a slip with the participant’s allocated group will be kept inside the envelope. After completing all baseline assessments, the research staff will provide the eligible participant with the sealed envelope. The participant will open the envelope and then be allocated to a group according to the slip inside. The opened envelope and the printed slip will be attached to the participant’s CRF (case report form) for record and trial monitoring. The timeline of the study is given in Table 1.

**Table 1.** Timeline of the study.

Content	Screening	Baseline	Day 14	Day 28	Day 56	Day 84	Day 112
Informed consent	✓						
Demographics and medical history		✓					
Assessment of eligibility criteria	✓						
Laboratory investigations	✓						✓
Ayurvedic parameters		✓	✓	✓	✓	✓	✓
Assessment of clinical parameters		✓	✓	✓	✓	✓	✓
Concomitant medication assessment			✓	✓	✓	✓	✓
Rescue medication assessment			✓	✓	✓	✓	✓
Assessment of ADRs^a^/AEs^b^			✓	✓	✓	✓	✓
Assessment of drug compliance			✓	✓			
Issue of trial/control drug and drug compliance report form		✓	✓				

a ADR: adverse drug reaction.

b AE: adverse event.

### Interventions

#### Pretreatment Period (Wash-Out Period):

There will be a wash-out period of two weeks only if the individual has a history of taking Allopathic/Ayurvedic/any medicine. Any oral or local application will be gradually withdrawn within a week, and the individual will not be given any medicine for the next week; then he/she will be registered for the trial.

### Drug Intervention

#### Group 1

Participants in Group 1 will be managed with the internal administration of 1000 mg *Triphala guggulu* and 6 gm of *Avipattikar churna* twice daily (BD) before food along with sitz bath in *Triphala kwatha* and *Jatyadi taila* application twice daily for 28 days. From day 14 to day 20, anal dilatation will be performed, followed by infiltration of 50 ml of *Jatyadi taila* for 7 days. Participants will be followed up to day 112. Details of the trial medicine in group 1 are shown in Table 2.

**Table 2.** Details of the trial medicine in group 1.

No.	Medicine	Dose	Dosage form	Route of administration	Anupana	Time of administration	Duration of therapy	Packing
1.	Triphala Guggulu	1000 mg Bd	tablet (one tab contain 500 mg)	Oral	Water	Twice a day, 30 minutes before food	28 days	60 tablet
2.	Avipattikar Churna	06 gm Bd	Churna	Oral	Water	Twice a day, 30 minutes before food	28 days	6 gm churna
3.	Triphala Churna	20 gm 2 times	Churna	Sitz bath	—^a^	Twice a day	28 days	20 gm churna
4.	Jatyaditaila	50 ml infiltration and also as lepana	Taila	External	—	Twice a day	28 days	450 ml

a Not applicable.

#### Group 2

Participants in group 2 will be treated with Diltiazem gel 2% for 28 days. They will be advised to apply the gel at least 1.5cm to 2 cm into the anus after a sitz bath in *Triphala kwatha*. During the trial period, participants will receive 6 gm of *Triphala churna* internally twice daily before food, and the follow-up will be done upto 112 days.

Lifestyle modification advice, including *Pathya-Apathya Ahara* and *Vihara,* will be given to all individuals in both group 1 and group 2. They will be advised to follow strict *Pathya Ahara – Vihara and* refrain from *Apathya ahara-vihara* (avoiding nonvegetarian food , *Katu-Amla Rasa and Ushna Veerya Aharas and Viharas* like continuous sitting, bike riding, etc) as per the *Rogavastha* and will be advised to consume foods rich in protein and dietary Fiber. Details of control medicine in group 2 are given in Table 3.

**Table 3.** Details of control medicine in group 2.

Sl.no.	Medicine	Dose	Dosage form	Route of administration	Anupana	Time of administration	Duration of therapy	Packing
1.	Triphala churna	6 gm BD	Churna	Oral	Water	Twice a day, 30 minutes before food	28 days	6 gm churna
2.	Triphala churna	20 gm 2 times	Churna	Sitz bath	—^a^	Twice a day	28 days	20 gm churna
3.	Diltiazem gel 2%	—	Gel tube	Individuals will be instructed to apply the gel at least 1.5 cm to 2 cm into the anus	—	Morning and night after sitz bath	28 days	30 gm

a Not applicable.

All the drugs will be procured from a GMP (Good Manufacturing Practices)-certified pharmacy at the Central Ayurveda Research Institute, Jhansi, India, as per the respective standards in the Ayurvedic Pharmacopeia of India (API) for this trial. The trial drugs will be stored in a dry, cool place, away from direct sunlight, high temperature, and strong-smelling substances like disinfectants and paints.

### Study Training

From Day 14 to Day 20, a Good Clinical Practices-certified doctor specialized in *Shalyatantra*, monitored by the medical team of the Principal and Co-investigators, will perform anal dilatation followed by anal infiltration with 50 ml of Jatyadi Taila. For female participants, the enrolment date will be scheduled so that the infiltration phase does not coincide with the menstrual cycle. After counseling, the participant will be positioned in the lithotomy position. Following adequate cleaning of the area, the expert will gently introduce a well-lubricated index finger, and then the index finger of the other hand will be introduced slowly using a crossed-hand technique. The fingers will be kept straight, and the sphincters will be gently stretched ihorizontally and held for 60 seconds. The procedure will be repeated in the vertical direction, again maintaining the stretch for 60 seconds [16]. During the infiltration phase in the subsequent days, the dilatation will gradually progress from two-finger to four finger dilatation. After gently introducing both middle fingers sequentially, the anus will be dilated again in each position, while maintaining the stretch for 60 seconds. Following completion of dilatation, anal infiltration with 50 ml of Jatyadi Taila will be administered. A pressure bandage will then be applied. The patient will be advised to lie down in the postoperative room for 30 minutes.

### Interim Analysis

An interim analysis, if required, would be conducted when at least 25% of participants have completed their the 16-week trial period.

### Outcome Measures

The primary outcome of the study is the assessment of reduction in the rate of recurrence of *Parikarthika*. Recurrence is defined as the percentage of participant-reported symptoms of fissure in ano after confirmed healing, validated by physical examination at the subsequent visits on the 56th, 84th, and 112th day after management with *Ayurveda* protocol, compared to the Diltiazem topical application. Day 112 will be considered the primary endpoint for final outcome assessment.

The secondary outcome will include the assessment of treatment efficacy of management protocol evaluated using the REALISE (The Scoring System for Anal Fissure) score, by degree of healing (fissure re-epithelialization), scoring system, and by spasm of anal sphincters in comparison with Diltiazem topical application. The investigators will record the parameters on the 14^th^, 28th, 56th, 84^th^, and 112^th^ day visits. Another secondary outcome will be the assessment of the safety of the *Ayurveda* management protocol regimen through reported incidence of adverse events recorded on the 14th day, 28th day, 56th day, 84th day, and 112^th^day and also comparing laboratory safety parameters (ie, CBC, complete blood count; LFT, liver function test; KFT, kidney function test) from baseline and 112^th^ day of follow up. Secondary outcomes will also include the assessment of comparative tolerability of the management protocol and Diltiazem topical application through reported incidence of adverse events during the study period recorded on the 14th and 28th days of visit. The study also assesses the change in health-related quality of life from baseline, assessed through the SF-12 (12-Item Short Form Health Survey) questionnaire and patient satisfaction rated on a scale of 0 (failure) to 5 (excellent) on the 14th day, 28th day, 56th day, 84th day, and 112^th^ day.

### Adherence

To ensure strict adherence to the protocol, Drug Compliance Forms were given to participants, and they were advised to fill and return the form at the time of the next visit. Additionally, the empty drug sachets, tubes, and bottles were collected from the participants to ensure compliance. Regular trial monitoring would be proposed for ensuring strict adherence to the protocol.

### Adverse Events

It includes any untoward medical occurrence that may present during treatment with a pharmaceutical product but does not necessarily have a causal relationship with this treatment.

### Adverse Drug Reaction (ADR)

It is a response that is noxious and unintended, occurring at doses normally used in humans for the prophylaxis, diagnosis, or therapy of disease, or for the modification of physiological function (WHO, 1972). An adverse drug reaction, contrary to an adverse event, is characterized by the suspicion of a causal relationship between the drug and the occurrence (ie, judged as being at least possibly related to treatment by the reporting or a reviewing health professional).

Any adverse event, if observed during the treatment period or during follow-up visits, should be clearly documented, and its appropriate and timely management should be undertaken. The Investigator should report the same to the Ethics committee and the sponsors at the earliest.

### Drug Compliance

If there is at least 80% compliance, the participant will be continued in the trial. Compliance needs to be assessed at each visit during the follow-up (ie, Day 14th and 28th) by counting the number of empty containers returned, and estimating the approximate number of capsules consumed by the individual.

### Concomitant Medication

A concomitant medication (con-med) is a drug or biological product, other than a study drug, taken by an individual during a clinical trial. Participants registered under the trial will be issued treatment cards with the entire treatment regimen written on them. They will be instructed to avoid using any other drugs on their own for any ailment, and will be clearly instructed to consult the treating Investigator for any symptom or complaint, or if they experience anything unusual. The Investigator will record any medications that he/she may prescribe to alleviate their ailments.

### Rescue Medication

Rescue medication/quick-acting medication/Fast-acting medication is a medication intended to relieve symptoms immediately when the symptoms worsen or breakthrough symptoms occur despite ongoing treatment. This contrasts with preventive medications, which are taken over a long period to prevent or manage symptoms. To alleviate any emergency, the use of rescue medication is permitted as per the wisdom/discretion of the Investigator. However, the same needs to be documented in an appropriate column in the CRF.

### Sample Size Determination

The recurrence rate of fissure in ano with nonsurgical procedures is 60%, and with the *Ayurveda* protocol, the expected recurrence rate is 30%. Considering a 5% level of significance and 80% power, along with the above inputs, the calculated sample size is 84 (42 in each group). Considering 5% dropout during the study, the modified sample size will be approximately 90 (45 in each group) [17].

### Drop-Outs

An attempt shall be made to record the reason for drop-outs, if any during the clinical trial.

### Data Collection

#### Baseline Assessment

At this visit, general information (including personal identification and demographic profile) will be recorded, medical history will be obtained, and general, physical and systemic examinations will be conducted. Subjective and objective parameters, as well as *Ayurvedic* parameters, will be assessed. Study medications will be dispensed, drug compliance will be reported through the issueance of drugs and Drug Compliance Report Form, and participants will be instructed to return for follow-up after 14 days.

#### Treatment Log

Physical examination, clinical assessment (including evaluation of subjective and Ayurvedic parameters), and assessment of drug compliance will be conducted on the 14th and 28th days of the treatment period. In addition, on the 14th day, drugs and the Drug Compliance Report Form will be issued. Group 1 participants will be admitted for dilatation and infiltration for 7 days and group 2 participants will be instructed to continue gel application. All the participants, irrespective of the group, will be advised to follow the *Pathya Ahara Vihara* as per the protocol

#### Follow Up Assessment

All participants will attend on the 56th, 84th, and 112^th^ days for follow up. Physical assessment, assessment of subjective and objective parameters, assessment of *Ayurvedic* parameters, and laboratory examination (on 112th day only) will be performed on all visits.

#### Data Documentation and Analysis

Data will be collected using paper and Electronic CRFs by the recruited Senior Research Fellow (SRF) and periodically checked by the Principal Investigator. The recorded data will be sent to the nodal team every month. All information regarding clinical trials should be properly documented, carefully handled, and meticulously stored to ensure accurate interpretation and verification. Analysis will be done by CCRAS’s Central Biostatistical Monitoring Unit. After completion of the study, the data will be stored for five years.

### Statistical Methods

After verification for accuracy, all collected data will be anonymized prior to statistical analysis. Any missing or incomplete responses will be addressed through imputation; to make the imputation robust, regression-based imputation will be applied if sufficient information is available in the data. Additionally, Bayesian imputation with an objective prior is our primary method for imputating the data. Further, the data will be used for descriptive analysis. Categorical variables will be described as frequencies and percentages, while continuous variables will be summarized as mean (SD)/median (Q1, Q3) depending on the distribution of data (normal/skewed). The inferential analysis will be performed after descriptive analysis of data; for within-group comparisons, data will be analyzed using the paired *t*-test/Wilcoxon sign rank test as per the distribution of the data for the pre-post observation, The repeated measures ANOVA/Friedman test will be used for within-group analysis for more than one follow-up. The between-group comparisons will be performed by the independent sample *t*-test/Mann-Whitney Test depending on the normality of the data. The generalized estimating Equation and rANOVA (Repeated Measures Analysis of Variance) analysis will also be performed if any confounding factors are found during analysis. The main analysis model for the primary outcome is ANCOVA (Analysis of Covariance), which will be performed if a baseline difference between groups is observed. All analyses will follow both the Intention-to-Treat (ITT) and Per-Protocol (PP) principles to evaluate the robustness and sensitivity of the results. A significance level of 5% will be considered for all analyses. All statistical analyses will be performed using STATA software (version 16) [18].

### Deviation From the Protocol

The trial should be conducted in compliance with the protocol. Deviations from the protocol should be made only when necessary to alleviate an immediate hazard to trial participants. All deviations from the protocol, including unplanned changes to interventions, examinations, data collection, and method of analysis, should always be reported to sponsors and IEC at the earliest, along with the exact reason for the deviation.

### Trial Monitoring

CCRAS’s Central Biostatistical Monitoring Unit (CBMU) and the technical officers who are directly involved in this project will monitor the progress of the trial through regular site visits. The purpose of these visits would be to ensure strict adherence to the trial protocol, correct completion of the forms, and to discuss any problems being faced by the research staff at the participating site.

Data Safety Monitoring Board (DSMB) will also be established according to the World Health Organization guidelines.

### Ethical Considerations

The trial will be conducted in accordance with ethical principles and guidelines. The Institutional Ethics Committee at NARIP (NARIP IEC: -17/07/24, F.No.03/01/2024/NARIP/Tech/786 dated 17.07.24) approved this study. The study has been registered in Clinical Trials Registry-India (CTRI) (CTRI/2024/09/073793). The study participants will be compensated for any financial losses with incidental support of INR 300 per visit. All study participants are covered under clinical trial insurance (National Insurance Company Limited, Invoice No.240400/24-03/12 dated 09/12/2024). This protocol is prepared as per the Standard Protocol Items: Recommendations for Interventional Trials (SPIRIT) statement [19]. Results will be published in a peer-reviewed journal with authorship eligibility according to the International Committee of Medical Journal Editors Criteria.

#### Individual Information and Consent Form

Prior to any trial-related activity, the Investigator will give the individuals verbal and written information about the trial in a form the participant can read and understand. The Investigator will ensure that the participant is fully informed about the aims, procedures, discomforts, and expected benefits. It must be emphasized that participation is voluntary and participants have the right to opt out of the trial at any time without any prejudice. Voluntary, signed, witnessed, informed consent should be obtained from the participant prior to any clinical trial-related procedure.

#### Confidentiality

Participant identities were kept confidential by assigning a unique identification code comprising the participant code, project code, and screening number. The actual identifying information was maintained separately in a master document to ensure confidentiality of data.

## Results

This project was funded in June 2024, and the study duration is 36 months. The first participant was enrolled in April 2025; 9 participants were enrolled as of May 2025. The data analysis will be completed by October 2027, and the results are anticipated to be published by December 2027.

## Discussion

This randomized controlled trial is designed to evaluate the efficacy of an ayurvedic management protocol in participants with *Parikarthika* (fissure in ano) with particular emphasis on recurrence, healing, and safety outcomes. A review article published in 2016 reported that conservative management strategies result in healing in up to 70% of patients, although these approaches are frequently accompanied by a high recurrence rate [8]. Notable adverse effects of these topical agents include recurrence, headaches, and toxicity [20]. Though lateral internal sphincterotomy is considered the gold standard treatment for chronic anal fissure, its potential complications, including flatus and fecal incontinence, necessitate the exploration and use of alternative pharmacological therapies [21]. In this view, the development of a well-structured Ayurvedic management protocol in chronic fissure in ano is highly relevant. Evidence from a randomized controlled trial indicates that Diltiazem provides better therapeutic outcomes in the management of chronic anal fissure, particularly in terms of enhanced healing and greater symptom reduction, when compared with glyceryl trinitrate [22]. Hence, topical Diltiazem application has been considered an appropriate comparator for evaluating the efficacy of the proposed management protocol. This study hypothesizes that participants receiving the *Ayurvedic* management protocol (group 1) will have a lower recurrence rate compared to those treated with topical Diltiazem. It will further assess the efficacy of the protocol in healing *Parikarthika* and evaluate its safety profile in comparison with topical Diltiazem application. In addition, comparative tolerability and changes in health-related quality of life will also be evaluated.

### Anticipated Results

Enrolment commenced in April 2025, and the study findings are expected following completion in October 2027. This trial will rigorously evaluate the efficacy of the *Ayurvedic* management protocol on the rates of recurrence, healing and safety in *Parikarthika* (fissure in ano), in comparison with topical application of Diltiazem. It is anticipated that the research protocol will demonstrate a significant reduction in recurrence rate compared to diltiazem application. The findings are expected to substantiate the development and implementation of a safe, effective, and structured management protocol for the comprehensive management of *Parikarthika*. The study results will be disseminated through peer-reviewed journals and scientific conferences.

### Strength

This study is the first RCT to evaluate the efficacy of an *Ayurvedic* management protocol for *Parikarthika* (anal fissure), with a particular focus on recurrence rates, healing outcomes, and safety. The outcome measures of this clinical trial will establish a robust database, that serves as a valuable foundation for future research. If the study yields positive results, it will help establish a standardized and structured protocol for the clinical management of *Parikarthika* (anal fissure).

### Limitations

A trained professional is essential for the proper implementation of this protocol. The study was designed to be conducted at a single center, focusing on a specific group of individuals from a single locality. A multicentre approach is recommended to evaluate whether the expected results are applicable to a diverse patient population. If the study is multicentric with a larger sample size, it will yield a better acceptance of the results.

### Conclusion

If this clinical trial demonstrates effectiveness, the proposed management protocol could be recognized as a standard potential approach for chronic anal fissure. Its incorporation into clinical practice may offer a cost-effective, efficient treatment option with a reduced recurrence rate, better outcomes, and safety.

## Supplementary material

10.2196/88548 Checklist 1 SPIRIT checklist.

## Abbreviations

ADR: Adverse Drug Reaction
AE: Adverse Event
ANCOVA: Analysis of Covariance
BD: Bis in Die (Twice daily)
CBC: complete blood count
CBMU: Central Biostatistical Monitoring Unit
CCRAS: Central Council for Research in Ayurvedic Sciences
CRF: Case Report Form
CTRI: Clinical Trials Registry-India
DSMB: Data Safety Monitoring Board
GMP: Good Manufacturing Practices
IEC: Institutional Ethics Committee
KFT: kidney function test
LFT: liver function test
NARIP: National Ayurveda Research Institute for Panchakarma
OPD: Out Patient Department
rANOVA: Repeated Measures Analysis of Variance
RCT: randomized controlled trial
REALISE: The Scoring System for Anal Fissure
SAE: Severe Adverse Event
SF-12: 12-Item Short Form Health Survey
SNOSE: Sequentially numbered opaque sealed envelopes
SPIRIT: Standard Protocol Items: Recommendations for interventional trials
SRF: Senior Research Fellow
